# Speech-generating devices: effectiveness of interface design—a comparative study of autism spectrum disorders

**DOI:** 10.1186/s40064-016-3181-6

**Published:** 2016-09-29

**Authors:** Chien-Hsu Chen, Chuan-Po Wang, I-Jui Lee, Chris Chun-Chin Su

**Affiliations:** 1 Ergonomics and Interaction Design Lab, Department of Industrial Design, National Cheng Kung University, No. 1, University Road, Tainan City, 701 Taiwan, ROC; 2 Department of Animation and Game Design, Shu-Te University, Kaohsiung, Taiwan, ROC

**Keywords:** Autism spectrum disorder, Communication disorders, Multiple-treatment reversal design, Speech generating device, Abbreviation-expansion menu

## Abstract

**Background:**

We analyzed the efficacy of the interface design of speech generating devices on three non-verbal adolescents with autism spectrum disorder (ASD), in hopes of improving their on-campus communication and cognitive disability. The intervention program was created based on their social and communication needs in school. Two operating interfaces were designed and compared: the Hierarchical Relating Menu and the Pie Abbreviation-Expansion Menu.

**Methods:**

The experiment used the ABCACB multiple-treatment reversal design. The test items included: (1) accuracy of operating identification; (2) interface operation in response to questions; (3) degree of independent completion. Each of these three items improved with both intervention interfaces.

**Results:**

The children were able to operate the interfaces skillfully and respond to questions accurately, which evidenced the effectiveness of the interfaces.

**Conclusions:**

We conclude that both interfaces are efficacious enough to help nonverbal children with ASD at different levels.

## Background

Non-verbal ability in association with autism spectrum disorder (ASD) disables the patients expressing basic wants and needs, making communication very difficult. Ganz et al. (2013) reported that approximately 20–30 % of those with ASD lack functional communication. About one third to one half of autistic adults and children are non-verbal, and have difficulty in language use, comprehension, and listening (Beukelman and Mirenda 2013; Cafiero and Meyer 2008; Carr and Felce 2007; Mirenda 2003). To overcome the communication barriers and improve the communication skills of children and adolescents with ASD, the augmentative and alternative communication (AAC) system has been adopted in the special education program, especially for those with severe communication disorders, allowing them to express themselves within minimal learning span.

Among the AAC systems, speech-generating devices (SGDs) [also known as voice output communication aids (VOCAs)] are a type of electronic aids that can support or replace language and writing by indicating images or sounds. The users can press the buttons on the SGD interfaces to facilitate communication with others (Beukelman and Mirenda 2013; Schlosser et al. 2009).

Numerous studies have explored the effect of AAC systems on social interaction, cognition, and assessment. Flores et al. (2012) reported that SGD and iPad interactive game could enhance social interaction and promote cognitive development and communication behavior among peers. This demonstrates the advantage of SGD intervention. (Cannella-Malone et al. 2009; Olive et al. 2007; Sigafoos et al. 2011, 2013; Trottier et al. 2011).

Boesch et al. (2013), Flores et al. (2012), and Sigafoos et al. (2009) compared PECS, SGD picture cards, and iPad usage. They found that all of those tools could enhance social interaction and natural language output, however, without significant difference among them. A further analysis into the PE, PECS, SGD, and other AAC system interventions found significances in the proximity and continuity aspects of social interaction.(Sigafoos et al. 2013; van der Meer et al. 2012). The reasons for the differences may be related to interface design and teaching methods.

Wallace et al. (2010), Gregory et al. (2006), and Bruno and Trembath (2006) studied the effects of image contextualization and verbal prompts on the accuracy and speed of the AAC navigational competence of users. With high- and low-context graphical interfaces at different levels, the users demonstrated significant differences in their cognitive abilities. They thus concluded that sentence length influences the level of improvement. Kagohara et al. (2012) used systematic instructional procedures and SGDs to encourage students with limited speech ability to engage in the educational activities. Wisenburn and Higginbotham (2008) examined the text pages of the dialogue interface, and found that phrases can achieve faster communication speed. However, for high- or low-class level, drop-down interface, scalable alpha mode, or a long sentence, very few researches focus on the operating effectiveness of users with ASD.

Autism-related language disorder, including lack of sentence organization ability, makes it difficult for those with ASD to communicate with others  (Kurtcu 2012; Binger and Light 2008). However, few studies have explored the effectiveness of the interface designs (such as hierarchical menus, pull-down menus, message formulation and retrieval mechanisms, and content presentation methods) of PE, PECS, VOCA, and SGD in AAC systems (Boesch et al.  2013; van der Meer and Rispoli 2010; Sigafoos et al. 2009). Hence, the research into organizational design content is particularly important.

This study considered the social and communication needs in school, and designed the Hierarchical Relating Menu (HRM) and Pie Abbreviation-Expansion Menu (PAEM) interfaces for non-verbal adolescents with ASD, in order to help them communicate with and respond to others in school. The main purposes of this study are: (1) to explore the use of SGDs by non-verbal adolescents with ASD; (2) to compare the effectiveness of HRM and PAEM in operating identification; (3) to evaluate whether the intervention of HRM and PAEM can increase the users’ accuracy in question responses; (4) to assess the difference in independent completion rate of the two interfaces; and (5) to discuss whether the two interfaces could improve users’ communication ability.

## Methods

### Participants

The participants of this study were three 12- to 13-year-old adolescents with ASD, from Xin-Xing Junior High School in Tainan. First, a signed consent form was obtained from their parents. Their speech-language pathologists, special education teachers, and parents were invited to interviews with ASD experts before the study. The Wechsler intelligence scale for children (WISC) was used to measure their intellectual ability. Table 1 lists the performance, language skil

**Table 1:** Wechsler intelligence scale for children intelligence quotient (IQ) scores of the participants

Participant no.	Name	Age	VIQ	PIQ	FSIQ	FSIQ disability description
1	Xuan	13	46	55	53	Cognitive weak, short words
2	Yi	13	56	56	55	Cognitive weak, short words
3	Feng	12	55	65	62	Cognitive weak, short words

*VIQ* verbal intelligence quotient, *PIQ* performance IQ, *FSIQ* full scale IQ

ls, and verbal intelligence quotient (VIQ) of the participants.

### Setting

The intervention was one 3-h session per week for 6 months. The experimental training was 30–35 rounds. The interface content was based on three basic social needs: greetings, requests, and responses. In the baseline environment, the target behavior of each participant was assessed. When the target behavior of the first participant appeared to reach a stable level, the intervention began for that child, and the other two remained at the baseline. When the target behavior of the first participant improved and was stable, intervention for the second participant began, etc.

### Materials

This study used a tablet PC (ViewPad 10 Pro; ViewSonic Taipei, Taiwan) because it was portable and allowed us to edit the files we used for the study. Picture Master Language Software (PMLS) Pro (Yuanding international Unlimiter ATEL Inc, Taipei, Taiwan) was used for layout design. The interface supports various devices, including touch screens.

### Research design

**Fig. 1:**
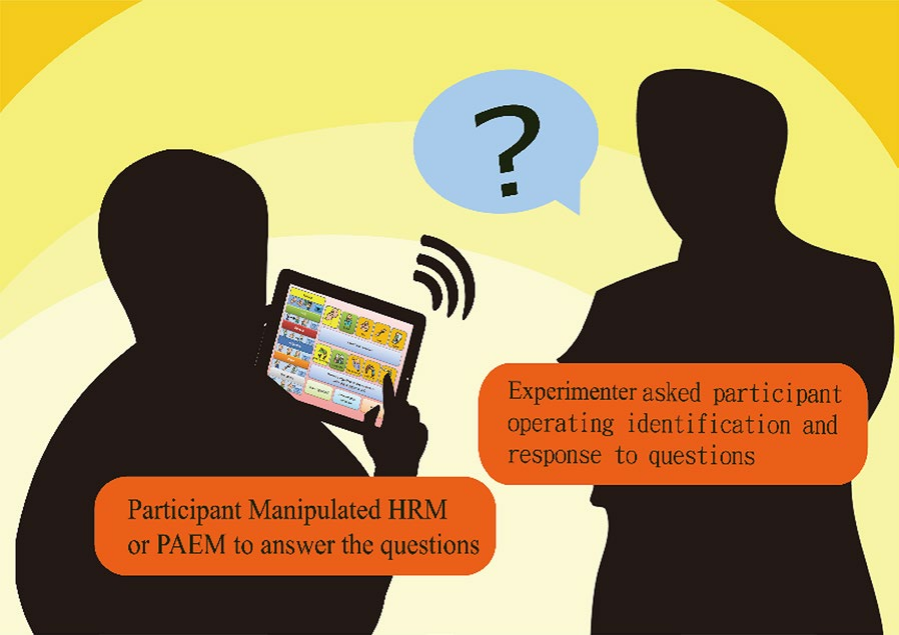
Training design for all participants

This study used the ABCACB Multiple-Treatment Reversal Design to evaluate the intervention outcome  (Kazdin 2011; Richards et al. 2013). The interface content was derived based on 20 types of campus scenarios. The following materials and strategies were used to train the participants to improve their semantic comprehension, simple grammatical organization, and vocabulary through social interactions (Fig. 1).

1. Hierarchical Relating Menu (HRM): This involved a connecting concept similar to the principle of typical Internet sitemap information. An HRM user can click on the basic content on the home page (Fig. 2) to connect to a desired second page based on its content and relevance.

**Fig. 2:**
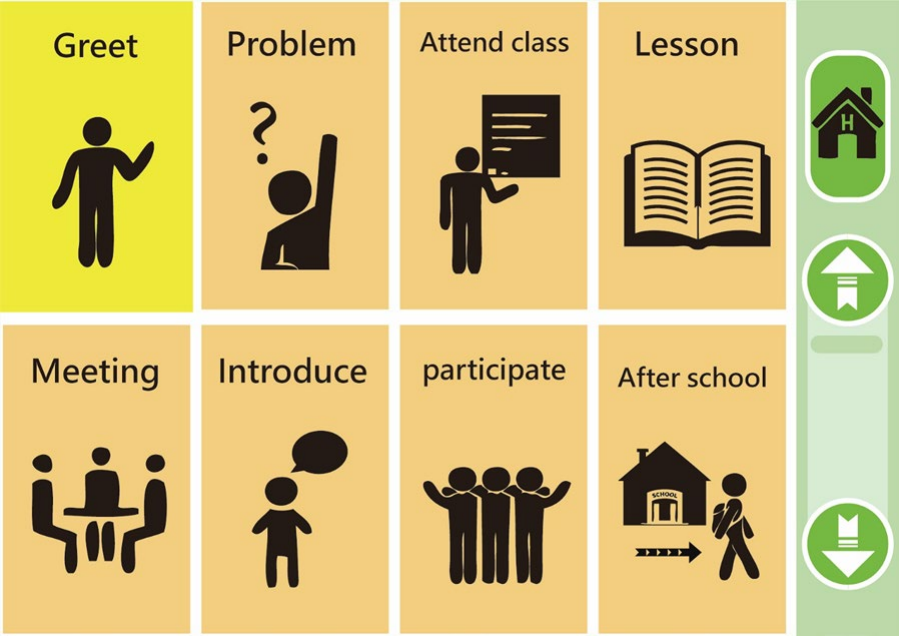
Hierarchical Relating Menu: Top-Page
2. Pie Abbreviation-Expansion Menu (PAEM): All of the content presented on the same page (Fig. 3) using a scalable storage mode screen legend and a vocabulary section.

**Fig. 3:**
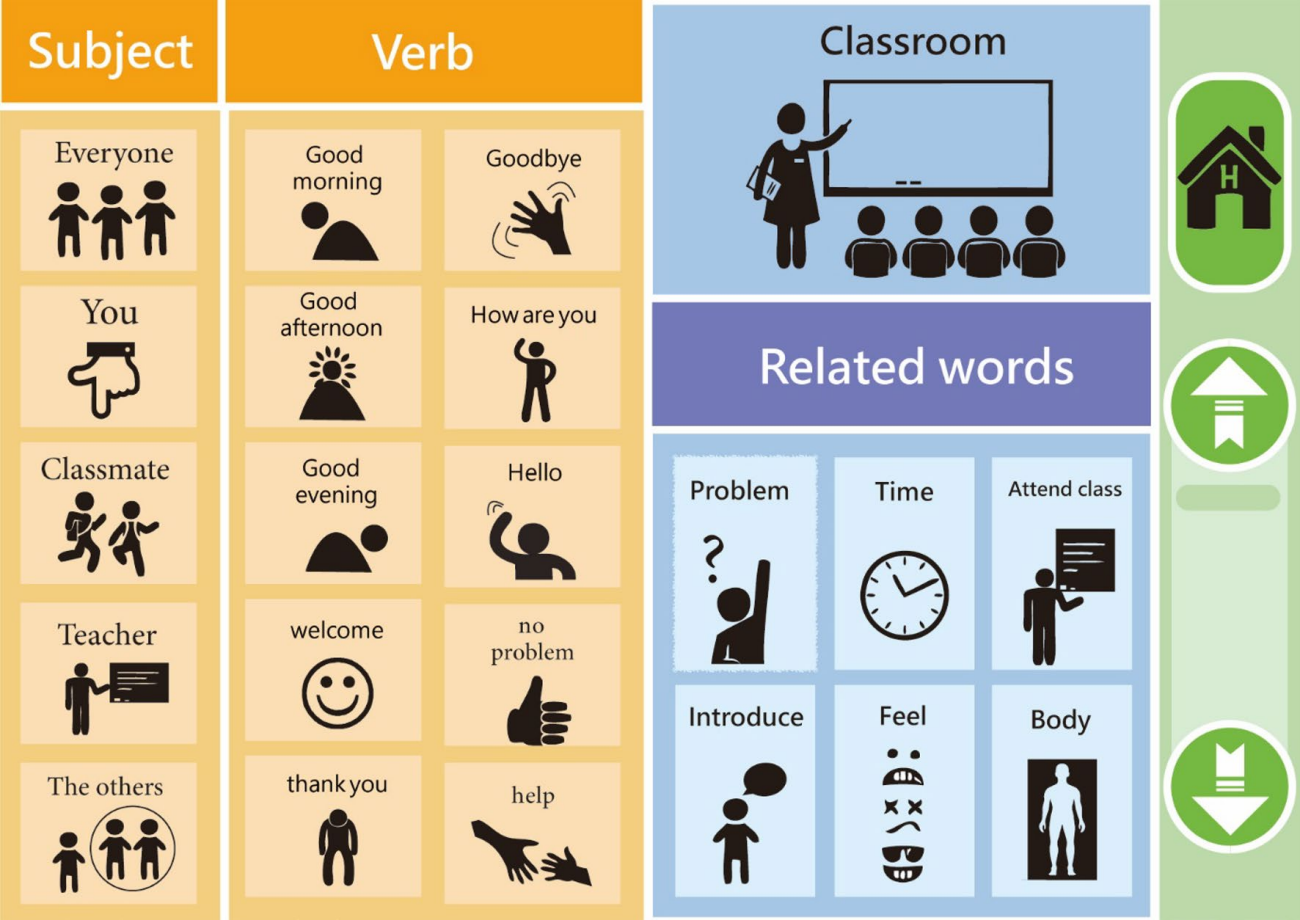
Hierarchical Relating Menu (HRM): Content-Page
3. Degree of completion of an independent assessment process: We set up an interface, based on an interface design evaluation system described elsewhere (Bailey and Wolery 1992), to prompt users to complete a specified task in order to determine the extent to which they had completed the operating process and the amount of assistance that they needed require. The levels of required assistance were defined as follows: complete independence (i.e., no assistance needed) = 10 points; verbal prompts needed = 8 points; assistance more substantial than verbal prompts (e.g., explanations, definitions, and encouragement) needed = 6 points; physical assistance (e.g.,) = 4 points; verbal instructions and limb assistance = 2 points; and an incomplete process = 0 points.

### Procedure

The training was provided in one 3-h session per week for 6 months. The participants were trained in the tutoring room. For all three participants, there were 3–7 sessions for baseline operation, 10 sessions for operating identification, 10 sessions for question responses, and 20 assessments of independent completion.

*Baseline (A1)*:

The baseline period did not involve any intervention, and focused only on the questions, such as common social questions and seeking help with questions, in order to find out whether the participants could identify expressive pictures, respond to questions, and reply (Appendix 1).

#### Intervention

*Treatment (B1)*:

In the HRM training session, the tasks involved five questions, and the participants were required to organize the communication content.

*Treatment (C1)*:

In the PAEM training session, the tasks involved five questions about classroom social conversation.

*Withdrawal (A2)*:

This stage returned to the baseline in order to confirm whether the intervention method had improved the communication and cognitive abilities of the participants. Using questions and answers, the researcher determined whether the participants could respond to questions using their own communication method.

*Treatment (C2)*:

In the second stage, the PAEM was used with the SGD for the same five questions asked in the first intervention stage.

*Treatment (B2)*:

The HRM was used with the SGD training session for the same five questions asked in the first intervention stage.

## Results

**Fig. 4:**
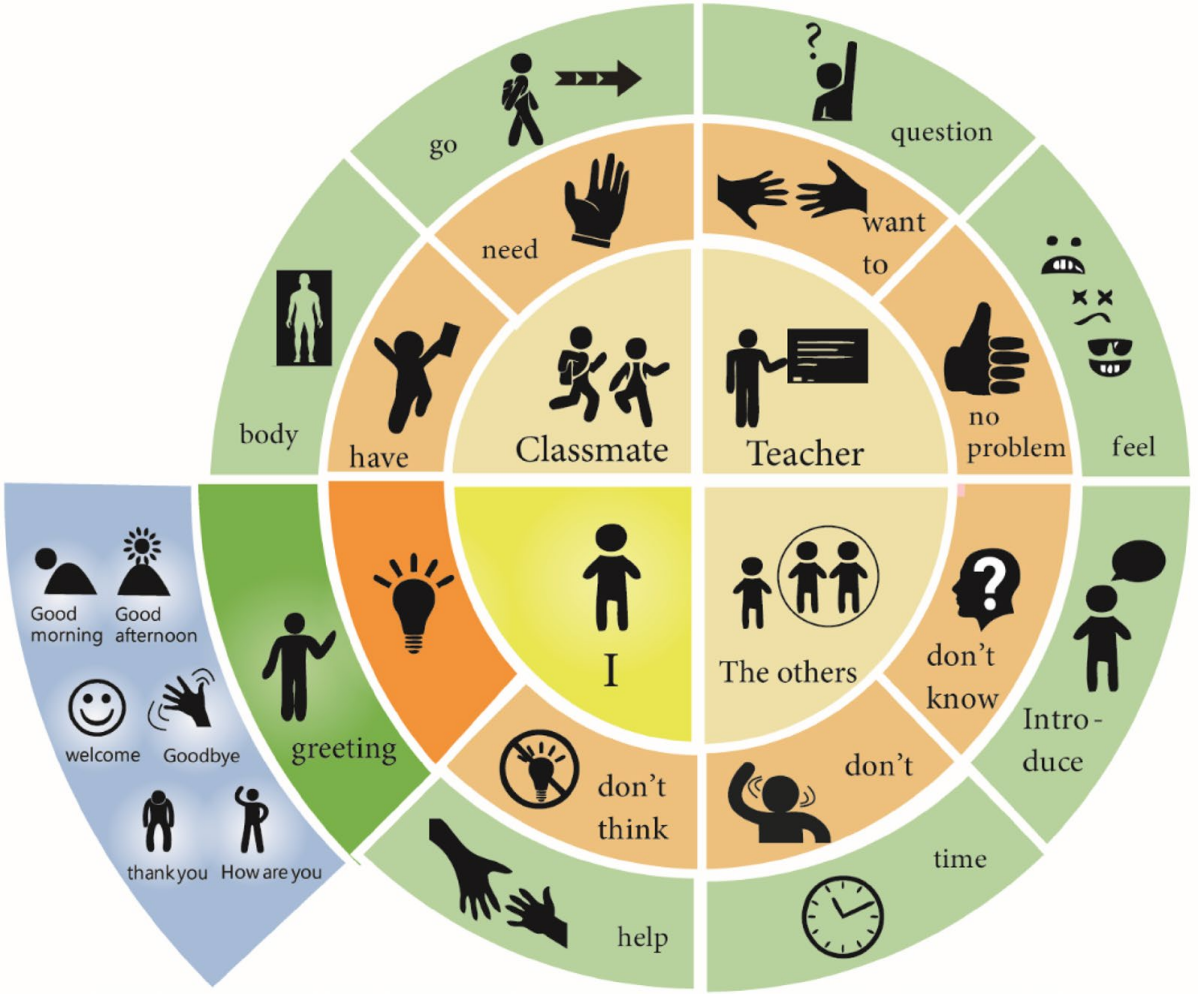
Pie Abbreviation-Expansion Menu (PAEM)-Circle style

For the first intervention stage, a Kruskal–Wallis H test was done to assess the effectiveness of the HRM and PAEM on SGD models, and to assess the improvement in communication and interaction behaviors in the two stages (Sigafoos et al. 2011). The Stage 1 experimental process is shown in Fig. 4. The Kruskal–Wallis H test showed that, for both interfaces, there were significant differences at baseline (A1), intervention B1, and intervention (C1) for all three participants (operating identification: χ^2^ [Participant 1: Xuan] = 7.454, p = 0.024); χ^2^ [Participant 2: Yi] = 12.828, p = 0.002; χ^2^ [Participant 3: Feng] = 13.822, p = 0.001) (Table 2). We therefore conclude that the two interfaces were effective for all three participants.

**Table 2:** Kruskal–Wallis, Mann–Whitney, χ^2^, and *p* values

Output condition	Phase 1:				Phase 2:			
	Operation-identification				Question-response			
	Kruskal–Wallis		Mann–Whitney		Kruskal–Wallis		Mann–Whitney	
	A1 B1 C1		B1 C1		A2 C2 B2		C2 B2	
	χ^2^	*p*	Z	*p*	χ^2^	*p*	Z	*p*
Xuan	7.454	0.024	−1.003	0.421	8.643	*0.013*	−1.826	0.095
Yi	12.828	*0.002*	−2.546	*0.008*	8.242	*0.016*	−1.571	0.151
Feng	13.822	*0.001*	−1.735	0.095	9.738	*0.008*	−2.293	*0.032*

Italic values indicate significant* p* values (*p* < 0.05)

In Stage 2, there were significant differences at baseline (A2), intervention (B2), and intervention (C2) for all three participants: (χ^2^ [Xuan] = 8.643, p = 0.013; χ^2^ [Yi] = 8.242, p = 0.016; χ^2^ [Feng] = 9.738, p = 0.008). This suggests that all three participants were able to respond to questions using the two interfaces.

Because the two independent sample *t* tests did not support the basic hypotheses, the Mann–Whitney test was used to compare the differences at interventions B1 and C1: Xuan: Z = −1.003, p < 0.421; Yi: Z = −2.546, p < 0.008; Feng: Z = −1.735, p < 0.095 (not significant) (Table 2). Only Yi showed a significant difference.

The analysis using variables B2 and C2 showed the following: Xuan: Z = −1.826, p < 0.095; Yi: Z = −1.571, p < 0.151 (insignificant); Feng: Z = −2.293, p < 0.032 (significant difference). The significant difference of B1-C1 for Yi (p = 0.008) and B2-C2 for Feng (p = 0.032) is possibly associated with personal preferences. Thus, the Wilcoxon signed-ranks test was also used to analyze the data.

**Fig. 5:**
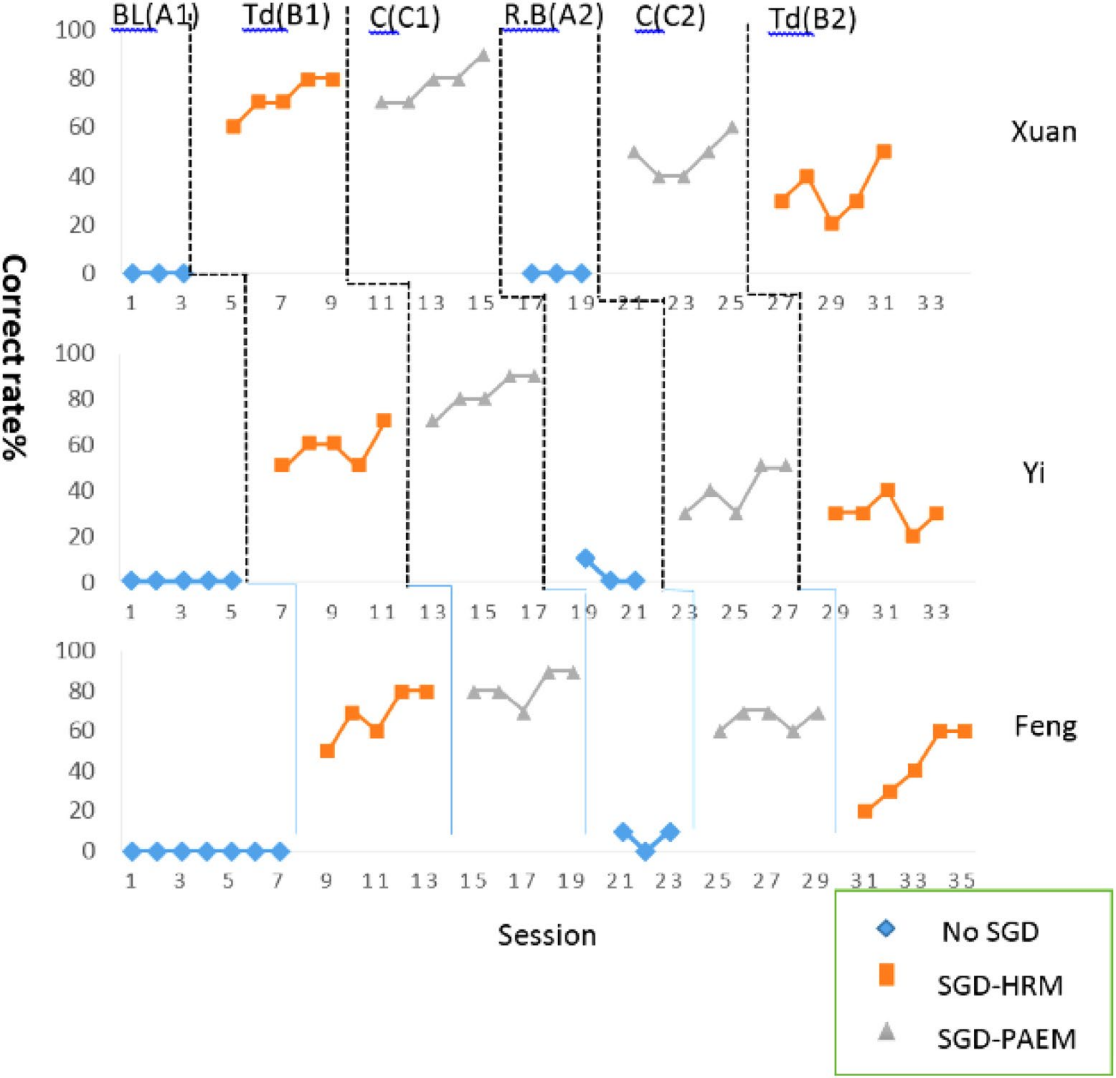
SGD operational definitions of the session

Except for the B2–C2 intervention, which led to a significant difference between Xuan and Feng in Stage 1, there were no other significant differences (Table 3). In Stage 2, there was a significant difference in the question response between Xuan and Feng, because Feng used the interface but Xuan did not (Fig. 4). The reason might be the differences in operation, especially when participants had to think before selecting the appropriate answers, which led to poor operating results. During the observation period, the level of independent completion of the HRM is was higher than that of the PAEM (Fig. 5). At Stage 1, the mean responses of the three participants using the HRM at B1–C1 were 7.2, 7.8, and 6.4, and at B2–C2 they were 6.6, 6.4, and 6.0, respectively. On research design levels of required assistance was between the stages of verbal instruction and instruction explanation. The completion rate of all three participants is consistently good. The completion rate of all three participants was consistently good.

**Table 3:** Wilcoxon, Z, and *p* values

Output condition	Phase 1:		Phase 2:	
	Operation-identification		Question-response	
Wilcoxon	B1-HRM		C2-PAEM	
	C1-PAEM		B2-HRM	
	Z	*p*	Z	*p*
Xuan versus Yi	−1.265	0.206	−1.387	0.165
Yi versus Feng	−1.414	1.57	−2.328	0.20
Xuan versus Feng	−0.000	1.000	−2.145	*0.032*

Italic value indicates significant* p* value (*p* < 0.05)

**Fig. 6:**
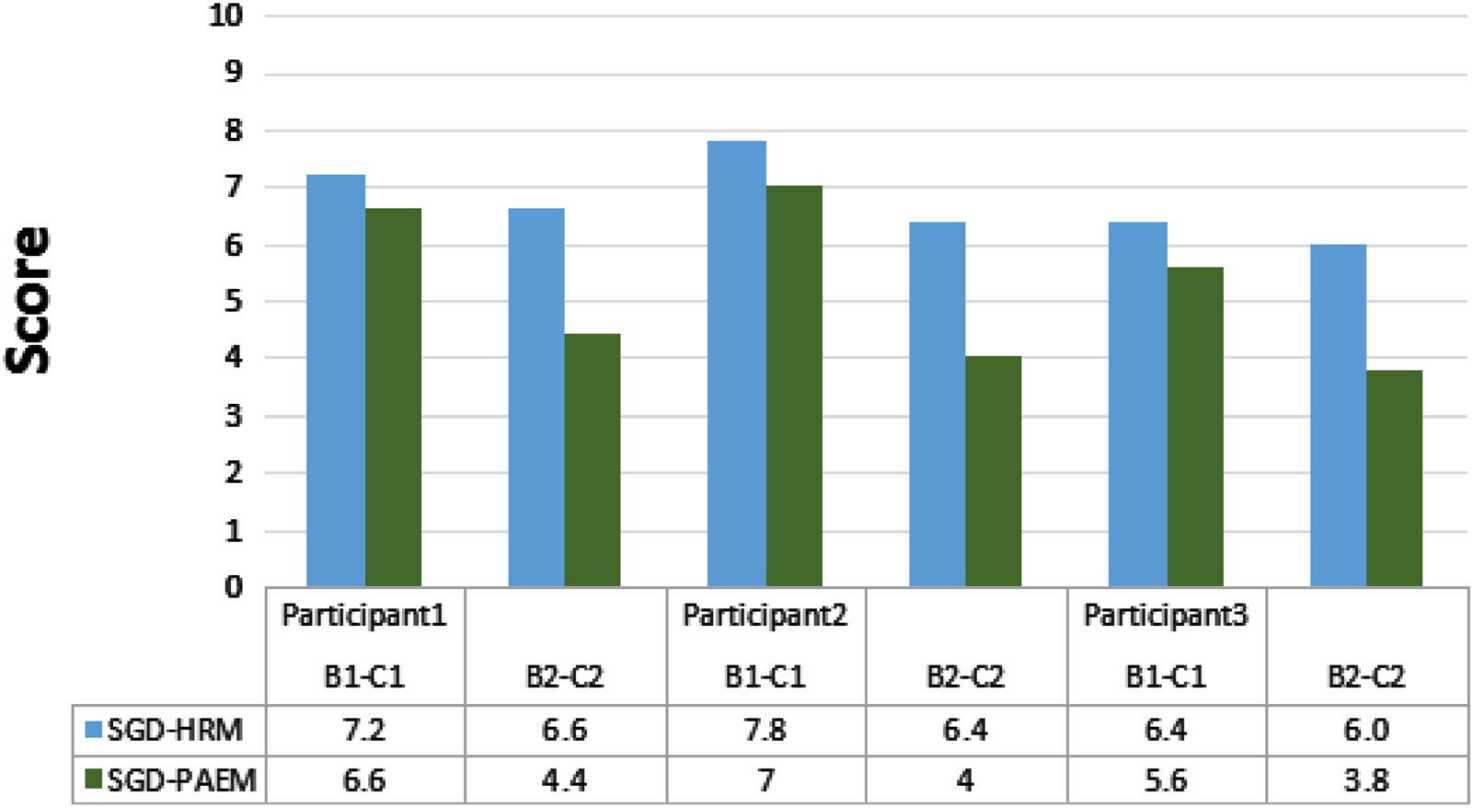
Degree of independent completion

For Stage 2, the participants’ level of independent completion is shown in Fig. 6. As seen, the mean of response of all three participants using the PAEM at intervention B1–C1 is 6.6, 7.0, and 5.6 and at B2–C2 is 4.4, 4.0, and 3.8, respectively. The results indicate that the operating identification of the participants at intervention B1–C1 is between verbal instruction and instruction explanation. At intervention B2–C2, more physical assistance and instructions might be required to complete the tasks.

## Discussion

Our major finding was that when our three nonverbal participants with ASD used the HRM and PAEM interfaces, they were able to communicate with others and even have simple conversations that used phrases like “Good morning!”, “How are you?”, and “Please help me.” The intervention of the two interfaces increased their operating identification and their ability to respond to questions. The participants’ accuracy in responses significantly improved. The completion time of each test indicated that the organizational pattern, length, complexity of sentences, and word order affect the intervention efficiency (Sigafoos et al. 2011; Wisenburn and Higginbotham 2008; Bruno and Trembath 2006;  Gregory et al. 2006).

The Kruskal–Wallis H test showed that there were significant differences in operating identification and question response after using the HRM and the PAEM. The operating identification and response accuracy of the three participants at B1-HRM and C1-PAEM were stable. Communication behavior and the training process both gradually progressed. On the contrary, the response accuracy of the participants during interventions C2-PAEM and B2-HRM regressed. The differences in the participants’ performances suggest that the interfaces still have room for improvement. For instance, the interface design, the order of operation, and the level of difficulty might limit the participants’ ability to respond.

The Mann–Whitney variables at B1 and C of Stage 1 show an independent distribution. There is no significant difference between Xuan and Feng. The reason that Yi is different might be that he had a preferred interface and more quickly learned to use that interface. An analysis of variables at C2 and B2 of Stage 2 also shows an independent distribution. There is no significant difference between Xuan and Yi. Feng’s question response performance indicates that the two interfaces have different effects, which might be a sign of personal preference.

When they used the PAEM, the three participants more accurately responded to questions. Although they learned to execute the command and operate the interfaces, operating proficiency when they had to respond to questions. Moreover, in both stages, the participants performed better in operating identification than in responding to questions. A comparison on operating effectiveness shows that, on average, interventions B1 and C1 resulted in greater accuracy, but that intervention B2 and C2 resulted lesser accuracy. Thus, the setting and operating sequence of the interfaces need to be improved. Our findings provide empirical evidence that clarify the findings of Kagohara et al. (2012). The levels of independent completion were good for all three participants, whether they used the HRM or the PAEM. This contradicts our finding that performance was better when the participants used the PAEM. The disagreement might be explained by how long it took each participant to decide to use the PAEM and by the complexity of the question. At intervention B1–C1, the level of assistance and promptness required for operating identification is low, while at B2–C2, more assistance is required when responding to the questions.

Our findings confirm that Xuan, Yi, and Feng found it difficult to respond to the questions regardless of the interface they used. These difficulties showed that more prompts are required for independent completion, for the following reasons: (1) participants need to choose an interface in order to complete the operating process, and (2) they need to understand the question in order to respond. The results showed a gap between the question responses and the operation of the interface. From this, a comparison of the interventions of B1–B2 and C1–C2, and an independent assessment of task completion, showed that the operating identification and the question response training, as well as the prompt process, are possible factors that influence the effectiveness of communication training.

Many studies have compared the effectiveness of different AAC devices such as PE, SGD, iPad, and Cyrano Communicator (Cannella-Malone et al. 2009; Sigafoos et al. 2009). Our findings are consistent with their finding that using SGDs helps people with ASD communicate with each other. Other studies have verified that, although there are significant differences between the two interfaces, comparisons of the training performance showed no completely similar preferred content. Those results support the notion that teaching has to be individualized (i.e., fitted to meet the learner’s specific needs), and that it is critical to modify the organizational interface used to strengthen teaching communication. The SGDs used in most of the studies on teaching simply displayed single-step and single-function (e.g., request) effects (van der Meer and Rispoli 2010). However, designing the difficulty level of the interface requires meticulous planning. The result of this study also supports the finding of Waddingtona et al. (2014) that systematic instruction is required.

## Conclusion

We found that, in Stage 1, the PAEM was more effective than was the HRM. This is because the PAEM interface places all the content on the same interface level, thus is easier to identify and operate. In Stage 2, a comparison of interventions B2 and C2 also showed that, for question response performance, the PAEM interface for question response performance was more effective than was the HRM interface. For independent completion, however, the HRM interface was more effective than was the PAEM interface, which indicates that the participants require different levels of assistance in Stage 2 than in Stage 1. The operating performance in Stage 2 was poorer, possibly because of (1) the participants’ familiarity with the interface; (2) their understanding of the questions; and (3) the complexity of the graphic layout, which affects their identification of the correct responses. This finding differs from Wallace et al. (2010), who claimed that training for the AAC interface is not required as long as the interface provides a sufficient number of graphic messages. On the contrary, the interface cannot be accurately operated unless a shallow-to-deep design is used and until the user becomes familiar with it. We therefore advise therapists to develop a sentence pattern classification and to organize in order to reduce the operating complexity, to allow SGD graphics to match the response interface, and train users how to use the SGDs.

According to Boesch et al. (2013) and Sigafoos et al. (2009), the intervention of PECS and SGDs affects behavioral changes, increases spontaneous communication skills, and improves participants’ social interactions. However, a comparison and assessment on the level of independent completion by the participants showed that they had to rely on different levels of prompts to complete various tasks. This suggests that, in order to facilitate the completion of tasks, a prompt interface and training program should be designed. Overall, the HRM and PAEM and the interventions do provide adolescents with ASD an effective alternative. Future studies should analyze the factors that affect operation, the conversion process, and question responses. Moreover, the effectiveness of the intervention provided by different mobile device content design might be investigated to further assess the definitions and rules. The interface design of sentence length affects the forms of voice output, which is consistent with Sigafoos et al. (2011). Therefore, the directions of different lengths of voice output and the influence of the communicating peers are also worthy of research.

## Limitations

This study had some limitations. First, because of the small pool of nonverbal adolescents with ASD in Taiwan, only three highly heterogeneous participants were recruited. Second, Mirenda and Erickson (2000) hypothesized that the development of communication and adolescent mental function are strongly related, and the present study did not stratify the participants in IQ-level groups, which would have been statistically meaningless because there were only 3 participants; thus, those IQs might have affected the results.

## Figures and Tables

**Fig. 7:**
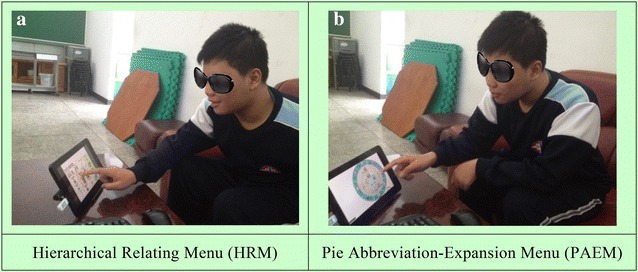
Sample Training**. a** Hierarchical Relating Menu (HRM), **b** Pie Abbreviation-Expansion Menu (PAEM)

## Appendix 1

See Table 4.

**Table 4:** Items for speech generating device training tasks

Title design/session	The correct rate/time					
	Pre	Post	Pre	Post	Pre	Post
	Xuan		Yi		Feng	
1 Social interaction—Hello!						
2. Social Interaction—Please do me a favor						
3. Social interaction—Pardon me?						
4. Social interaction—Good morning, teacher!						
5. Social interaction—I’m sorry. I pressed the wrong button.						
6. Help—May I borrow your notes?						
7. Help—I would like to borrow your homework.						
8. Help—Would you please wait for me?						
9. Help—I can’t hear you						
10. Help—I can’t understand						
11. Ready for class—What lessons are you going to?						
I’m going to physical education class						
12. Ready for class—What lessons are you going to?						
I’m going to math class						
13. Self-introduction—Hello, I’m.						
14. Self-introduction—Are you?						
15. Participation—What activities do you want to participate in?						
I want to participate in.						
16. Participation—What activities you want to participate in?						
I want to participate in						
17. Academics—Are you ready to do what you have to do?						
Are you ready to do your homework and go home?						
18. Academics—Did you prepare?						
19. After-school time—I can play with you now.						
20. After-school time—Where do you want to go? I want to go home						
Operation-Identification accuracy %						

## Appendix 2

See Fig. 7.
